# A New Machine Learning-Based Framework for Mapping Uncertainty Analysis in RNA-Seq Read Alignment and Gene Expression Estimation

**DOI:** 10.3389/fgene.2018.00313

**Published:** 2018-08-14

**Authors:** Adam McDermaid, Xin Chen, Yiran Zhang, Cankun Wang, Shaopeng Gu, Juan Xie, Qin Ma

**Affiliations:** ^1^Bioinformatics and Mathematical Biosciences Lab, Department of Agronomy, Horticulture, and Plant Science, South Dakota State University, Brookings, SD, United States; ^2^Department of Mathematics and Statistics, South Dakota State University, Brookings, SD, United States; ^3^Center for Applied Mathematics, Tianjin University, Tianjin, China; ^4^Department of Electrical Engineering and Computer Science, South Dakota State University, Brookings, SD, United States

**Keywords:** gene expression, RNA-Seq read alignment, mapping uncertainty, machine learning, elastic-net, mixture model fitting, k-means clustering, EM-algorithm

## Abstract

One of the main benefits of using modern RNA-Sequencing (RNA-Seq) technology is the more accurate gene expression estimations compared with previous generations of expression data, such as the microarray. However, numerous issues can result in the possibility that an RNA-Seq read can be mapped to multiple locations on the reference genome with the same alignment scores, which occurs in plant, animal, and metagenome samples. Such a read is so-called a multiple-mapping read (MMR). The impact of these MMRs is reflected in gene expression estimation and all downstream analyses, including differential gene expression, functional enrichment, etc. Current analysis pipelines lack the tools to effectively test the reliability of gene expression estimations, thus are incapable of ensuring the validity of all downstream analyses. Our investigation into 95 RNA-Seq datasets from seven plant and animal species (totaling 1,951 GB) indicates an average of roughly 22% of all reads are MMRs. Here we present a machine learning-based tool called ***GeneQC*** (**Gene** expression **Q**uality **C**ontrol), which can accurately estimate the reliability of each gene's expression level derived from an RNA-Seq dataset. The underlying algorithm is designed based on extracted genomic and transcriptomic features, which are then combined using elastic-net regularization and mixture model fitting to provide a clearer picture of mapping uncertainty for each gene. GeneQC allows researchers to determine reliable expression estimations and conduct further analysis on the gene expression that is of sufficient quality. This tool also enables researchers to investigate continued re-alignment methods to determine more accurate gene expression estimates for those with low reliability. Application of GeneQC reveals high level of mapping uncertainty in plant samples and limited, severe mapping uncertainty in animal samples. GeneQC is freely available at http://bmbl.sdstate.edu/GeneQC/home.html.

## Introduction

RNA-Seq is a revolutionary high-throughput process that allows researchers to observe the genetic makeup of a particular sample (Wang et al., 2009; Garber et al., 2011; Ozsolak and Milos, 2011) and can assist in determination of regulatory mechanisms and transcription unit prediction (Chou et al., 2015; Chen et al., 2017). Research involving RNA-Seq data produces gene expression profiles, in which a discrete expression value for each annotated gene for that species is identified. These gene expression profiles are extracted through computational analysis pipelines (Trapnell et al., 2009; Andrews, 2010; Wang et al., 2010; Grabherr et al., 2011; Kong, 2011; Li and Dewey, 2011; Dobin et al., 2013; Philippe et al., 2013; Wu et al., 2013, 2016; Anders et al., 2015; Bonfert et al., 2015; Chang et al., 2015; Kim et al., 2015; Pertea et al., 2015, 2016; Yuan et al., 2017), which can be analyzed further to identify differentially expressed genes between treatment groups (Robinson et al., 2010; Anders and Huber, 2012; Trapnell et al., 2012; Ritchie et al., 2015; Pimentel et al., 2017; Monier et al., 2018), enriched functional gene modules (Subramanian et al., 2005; Zhou and Su, 2007; Chen et al., 2009; Pathan et al., 2015), co-expression networks (Zhang et al., 2016; Cao et al., 2017), and to generate visualizations to assist in broad interpretations between treatment groups (Goff et al., 2013; Powell, 2015; Younesy et al., 2015; Ge, 2017; Harshbarger et al., 2017; Nelson et al., 2017; Nueda et al., 2017; McDermaid et al., 2018a; Perkel, 2018), among other applications.

One application of RNA-Seq analysis pipelines is to use the sequenced RNA-Seq reads (or *reads* for short) with a reference genome, if available, to estimate the expression level of each gene (Nagalakshmi et al., 2008; Miller et al., 2014). The basic process is to map these reads to the location with the best alignment score on the reference genome (Wu et al., 2014). Even though numerous methods have been developed to facilitate this analysis, some critical issues persist. The nature of DNA—long strands of millions of base-pairs created by a reordering of the four nucleotides—makes it inevitable that some similarities and duplications will occur throughout the genome. This can lead to ambiguity during read mapping, with specific reads being aligned to multiple locations across the reference genome with the same alignment scores (Li et al., 2009; Oshlack et al., 2010; Swan, 2013; Trapnell et al., 2013; Baruzzo et al., 2017).

This MMR problem can be observed in any genomic region, including, exons and transcripts. For conciseness, we refer to these genomic regions simply as “genes.” This issue has been observed in many diploid species, including human and other mammals and Arabidopsis (Albrecht et al., 2009; Cho et al., 2009; Yoder-Himes et al., 2009; Zhu et al., 2011; Network CGAR., 2018;), as well as many multiploid species (Consortium IWGS., 2014). In some species, such as *Glycine max*, up to 75% of the genes have the duplicated partners in its genome (Schmutz et al., 2010). For species with high levels of uncertainty, especially angiosperms, the MMR problem can have serious implications on gene expression levels and can be extremely hard to remediate due to the genes' and chromosomes' duplicative nature. To more fully investigate the prevalence of MMRs in current RNA-Seq analyses, we analyzed almost two terabytes of RNA-Seq data from seven plant and animal species. Upon analysis of this data, it was clear that a large amount of MMRs was present in a variety data. Thus, mapping uncertainty is inevitably affecting the gene expression estimates and eventually causing bias in downstream analyses.

During our initial investigation into the MMR problem, 95 datasets totaling 1,951 GB were analyzed. Both paired- and single-end reads were collected from NCBI (Coordinators, 2016), URGI (https://urgi.versailles.inra.fr/), and JGI (Nordberg et al., 2013) for seven plant and animal species. These species include *Arabidopsis thaliana, Vitis vinifera, Solanum Lycopersicum, Panicum Virgatum, Triticum Aestivum, Homo sapiens*, and *Mus musculus*. The 95 datasets average 20.6 GB, with an average overall alignment rate of 81.87%. Each dataset was aligned using HISAT2 (Kim et al., 2015) against the appropriate reference genome. Alignment statistics were collected or calculated from the HISAT2 output file, as shown in Table 1. It was determined that an average of 22% of all reads were ambiguously aligned in each of the seven distinct plant and animal species. In four datasets, over 35% of the reads were ambiguously aligned, and over two-thirds of the analyzed datasets having at least 18% of the reads multi-mapped. *Panicum virgatum* exhibited the highest overall proportions—ranging from 17 to 33%—of MMRs over all analyzed datasets, while *Arabidopsis thaliana* displayed the lowest proportion, ranging from 8 to 17%. The other analyzed species had similar percentages of MMRs. More details of the MMR analyses over these 95 datasets can be found in Supplementary File 1.

**Table 1:** Multi-mapped reads.

**Species**	***Arabidopsis thaliana***	***Vitis vinifera***	***Solanum lycopersicum***	***Panicum virgatum***	***Triticum aestivum***	***Homo sapiens* Genome**	***Homo sapiens* Transcriptome**	***Mus musculus* Genome**	***Mus musculus* Transcriptome**	**Total**
Datasets	10	10	10	10	13	11	11	10	10	95
Size(GB)	153.7	152.3	151.8	385.7	348.1	249.9	249.9	129.9	129.9	1,951
Unique-mapped	69–89%	55–82%	52–88%	47–66%	61–69%	56–71%	59–70%	41–73%	41–75%	55%
Multi-mapped	8–17%	9–25%	5–34%	17–33%	17–25%	16–27%	15–24%	9–37%	9–36%	22%
Un–mapped	2–17%	8–23%	4–16%	13–25%	9–18%	12–21%	12–22%	3–31%	2–31%	23%
(Multi-mapped)/(total mapped)	8–18%	10–31%	6–39%	22–39%	21–28%	19–32%	19–28%	11–47%	11–47%	29%

*The alignment statistics for the 95 analyzed datasets across seven species, indicating the ranges of percentages for the uniquely aligned, multi-mapped, and un-mapped reads, as well as the proportion of multi-mapped out of the total mapped reads*.

The general solution of the MMR problem in previous studies is to discard or evenly distribute to all potential locations, leading to severe, biased underestimation or overestimation of the gene expression levels, respectively (Kim et al., 2013). More commonly, a proportional assignment of ambiguous reads, in which the read is segmented in smaller portions based on the number of possible mapping locations and uniquely mapped reads to each of them (Li et al., 2009). Recently, additional methods have been employed to attempt remediation of mapping uncertainty after initial alignment (Li and Dewey, 2011; Kahles et al., 2015; Bray et al., 2016). However, even these realignment strategies do not provide a thorough method for evaluation of the alignment quality. While RNA-Seq pipelines traditionally begin with read-level quality control using FastQC (Andrews, 2010), no such method currently exists for controlling the quality of gene expression estimation after read alignment.

If researchers continue processing RNA-Seq data with such high levels of mapping uncertainty, all downstream analyses will have skewed and biased results. Just as raw reads require quality control (Andrews, 2010) so do gene expression estimates based on mapping results. Even with tools that are specifically designed to address mapping uncertainty, such as *MMR* (Kahles et al., 2015), the quality of the derived gene expression estimates based on mapping results still requires investigation, especially in real datasets not simulated datasets. Without some quality control for gene expression estimation, researchers could potentially be using unreliable data, and blindly doing so.

One promising method for addressing the issue of gene expression-level quality control is the implementation of machine learning. It uses or relates to following concepts or algorithms including statistics, artificial intelligence, philosophy, information theory, biology, cognitive science, computational complexity and control theory to give computers and algorithms the ability to learn and improve performance on a specific task without being explicitly programmed (Mitchell, 1997). Machine learning has two main categories: supervised and unsupervised learning. The majority of practical studies use supervised learning methods to train the relationship from the input to the output, using provided category labels or resultant values to develop a mapping function for the prediction of unlabeled data. Specifically, Elastic-net regularization, a supervised method, was used in this research. Meanwhile, machine learning can also be used to train a model from unlabeled data through the unsupervised learning, aiming to model the underlying structure or distribution in the training data for clustering and association problems. Two unsupervised learning algorithms were used in this study, i.e., K-means clustering and the Expectation-Maximization algorithm (EM-algorithm).

**Figure 1:**
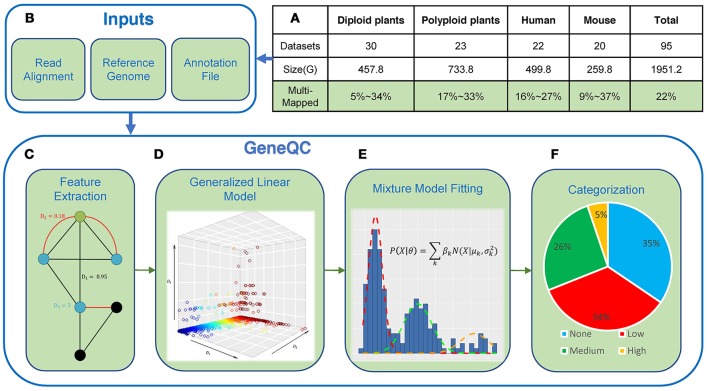
Mapping Uncertainty and GeneQC**. (A)** The MMR percentages for the 95 datasets across seven species. More detailed information is showcased in Table 1; **(B)** GeneQC takes a read alignment, reference genome, and annotation file as inputs; **(C)** The first step of GeneQC is to extract features related to mapping uncertainty for each annotated gene; **(D)** Using the extracted features, elastic-net regularization is used to calculate the D-score, which represents the mapping uncertainty for each gene; **(E)** A series of Mixture Normal and Mixture Gamma distributions are fit to the D-scores; and **(F)** The mixture models are used to categorize the D-scores into different levels of mapping uncertainty along with a statistical alternative likelihood value for each gene.

To address issue of mapping uncertainty, we present the machine learning-based tool GeneQC (Figure 1), which uses extracted multi-level features combined with novel applications of regularized regression and mixture model fitting approaches to quantify the mapping uncertainty issue (McDermaid et al., 2018b). This tool can determine the genes having reliable expression estimates and those require further analysis, along with a statistical significant evaluation of the mapping uncertainty level. GeneQC develops a novel score, referred to as D-score, to represent the level of mapping uncertainty for each annotated gene and groups genes into several categorizations with different reliability levels, through integration and modeling of three genomic and transcriptomic features. Specifically, (i) sequence similarity between a particular gene and other genes is collected to give an insight into the genomic characteristics contributing to the MMR problem; (ii) the proportion of shared MMR between gene pairs provides information regarding the transcriptomic influences of mapping uncertainty within each dataset; and (iii) the degree of each gene, representing the number of significant gene pair interactions resulting from calculating (i) and/or (ii). More details of the procedure can be found in the Methods section.

## Methods

### GeneQC implementation

GeneQC is designed to fit into computational pipelines for RNA-Seq data immediately following read alignment, acting as a supplement to most current pipelines. GeneQC is composed of two distinct processes: feature extraction and statistical modeling. The feature extraction process is implemented using a Perl program and the statistical modeling is performed on the feature extraction output using an R package, which provides the final output for GeneQC (http://bmbl.sdstate.edu/GeneQC/download.html). More details on the implementation of GeneQC can be found at http://bmbl.sdstate.edu/GeneQC/tutorial.html.

### Required inputs

GeneQC takes as inputs three pieces of information that are easily found in most RNA-Seq analysis pipelines: (1) the read mapping result SAM file; (2) the fasta reference genome corresponding to the to-be-analyzed species; and (3) the species-specific annotation general feature format (gff/gff3) file (Figure 1B). Example datasets can be found on the GeneQC webserver at http://bmbl.sdstate.edu/GeneQC/result.html.

### Feature extraction

**Figure 2:**
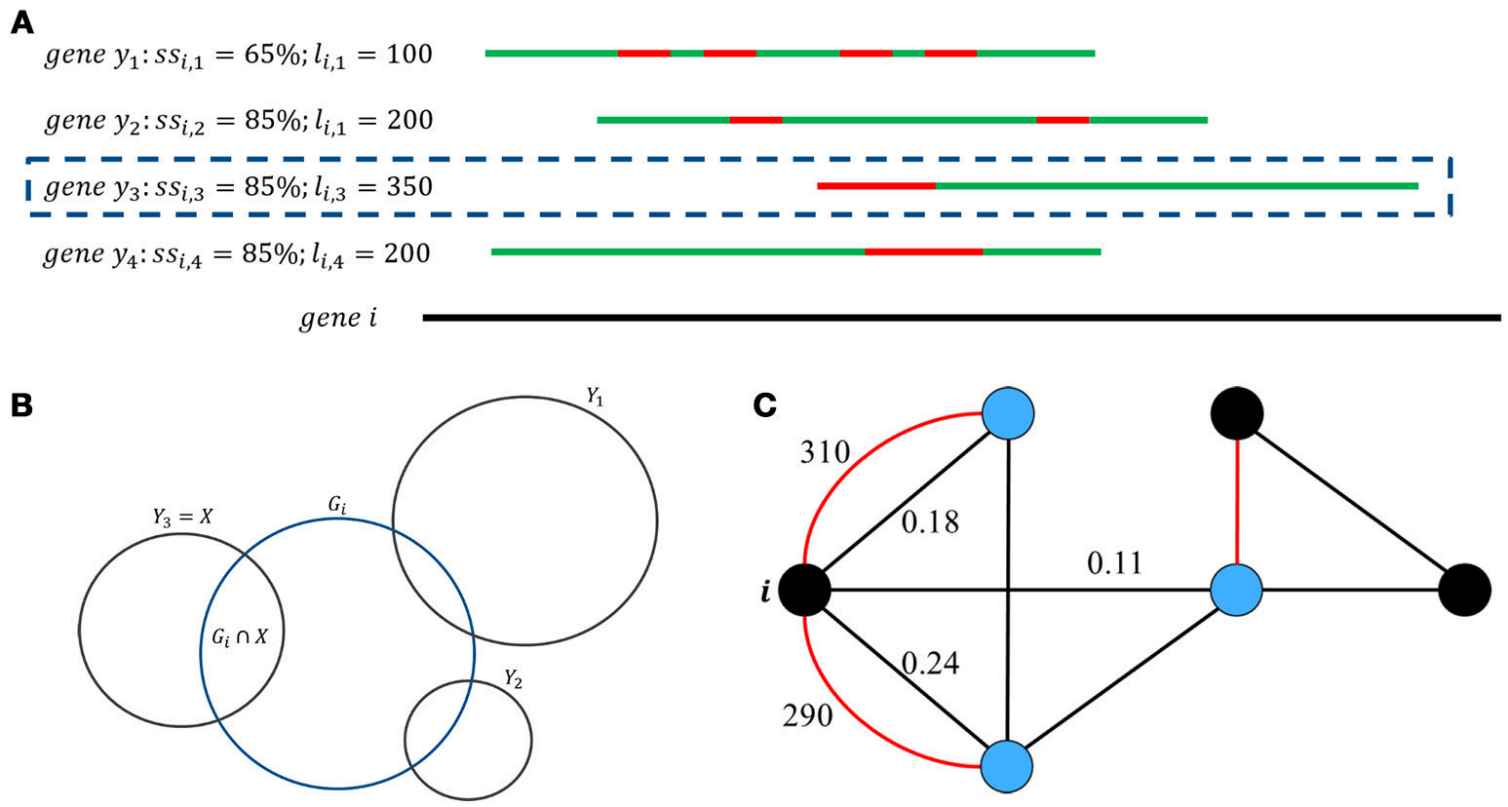
Genomic, transcriptomic, and network feature development. **(A)** Genes with significant similarity are displayed, with *D*_1_ being the maximum value of *ss*_i,y_^*^l_*i,y*_. In this situation, genes *y*_2_, *y*_3_, &*y*_4_ all have the same *ss*_*i*_ value, but gene *y*_3_ has a longer consecutive string of matching base pairs (*l*_*i*_) than the other values, making it the more similar genomic location. **(B)** Graphical representation of the sets of reads aligned to each gene. *D*_2_ is the largest overlapping proportion of shared ambiguous or multi-mapped reads between the target gene, gene *i*, and all other genomic locations that have at least one read potentially aligned to both locations. **(C)** This graph displays the significant interactions of gene *i* with other genomic locations. Each node represents a genomic location, with the red edges representing sequence similarity scores and black edges representing multi-mapping proportions. In this situation, *D*_1_ = 310, *D*_2_ = 0.24, and *D*_3_ = (3+1) = 0.602.

From input information, GeneQC first performs feature extraction, in which the three characteristics are calculated for each annotated gene (Figure 1C). The first extracted feature (*D*_1_) is derived from genomic level information and involves the similarity between two genes (Figure 2A). For each gene, this is calculated as the maximum of the sequence similarity multiplied by the match length, where the match length is the longest continuous string of matching base pairs. More specifically, $D_{1}=\underset{y}{\max}\left\{ss_{i,y}*l_{i,y}\right\},$ where *ss*_*i, y*_ is the base pair sequence similarity of gene *i* and gene *y* and *l*_*i, y*_ is the match length of these two genes.

The second feature (*D*_2_) comes from transcriptomic level information and represents the proportion of shared MMRs (Figure 2B). This value is calculated as the maximum proportion of shared MMRs between the gene of interest and another gene. In other words, $D_{2}=\frac{\left|G_{i}\cap X\right|}{\left|G_{i}\right|}$, where *G*_*i*_ = {*all reads aligned to gene i*} and *X* = |G_i_∩ Y |.

The third feature (*D*_3_) is a network factor that represents the number of alternate gene locations with significant interactions with the gene of interest based on the previous two parameters (Figure 2C) and is calculated as *D*_3_ = log_10_(|*S*^∪^*M*|+1), where *S* = {*genomic locations with D*_1_ > 0} and *M* = {*genomic locations with D*_2_ > 0}.

In addition to understanding the severity of the MMR problem in each sample, GeneQC provides species- or sample-specific insight into each feature's impact on mapping uncertainty. This is done by developing a linear model to determine the significance and degree of impact for each feature.

### GeneQC modeling

#### Dependent variable construction

To perform the modeling, a dependent variable is constructed. The dependent variable *D*_4_ is an approximation of the proportion of ambiguous reads based on the two most extreme approaches to dealing with multi-mapped reads, the unique alignment approach and the all-matches approach. If we consider *G*_*i*_ = {*reads mapped to gene i*} and *U*_*i*_ = {*reads uniquely mapped to gene i*}, the true alignment *R*_*i*_ must fall somewhere between these two values, with |*U*_*i*_| ≤ |*R*_*i*_| ≤ |*G*_*i*_|. Thus, we approximate the true alignment as $|{\hat{R}}_{i}|=\frac{\left|G_{i}|+|U_{i}\right|}{2}$. Using this approximation, we calculate

$$\begin{array}{l} D_{4}=1-\frac{\left|{\hat{R}}_{i}\right|}{\left|G_{i}\right|}=1-\frac{\left|G_{i}|+|U_{i}\right|}{2|G_{i}|} \end{array}$$

#### Elastic-net regularization

To develop a model evaluating the severity of mapping uncertainty and thus expression estimation quality, a regression approach is utilized. Ordinary least squares has been demonstrated to have particular issues when dealing with real world data, especially data that does not fit linearity, homoscedasticity, lack of serious multi-collinearity, or other requirements (Dempster et al., 1977). Because of this, alternative approaches were explored. Ridge regression, which develops a model based on an *L2-norm* penalization, has better predictive results than ordinary least squares regression (Hoerl and Kennard, 1970; Dempster et al., 1977). However, this approach tends to retain all included variables to achieve such high predictive power, in turn reducing the interpretability of the model (Zou and Hastie, 2005). Another approach with potential application in GeneQC is the least absolute shrinkage and selection operator, also known as lasso. This method uses an *L1-norm* penalization, while simultaneously performing continuous shrinkage and variable selection (Tibshirani, 1996). While this is an appealing feature in generating a model, lasso has shortcomings when it comes to dealing with variables exhibiting high pairwise correlation (Zou and Hastie, 2005). Elastic-net regularization—sometimes referred to simply as elastic net—has the potential to overcome the shortcomings of both ridge and lasso regression methods by implementing a combination of the two approaches.

Take the set of *n* response variables $\text{y}={\left(y_{1},\;y_{2},\ldots ,y_{n}\right)}^{T}$, a set of *p* predictor variables **x**_**i**_ = (*x*_*i*, 1_, *x*_*i*, 2_, …, *x*_*i, p*_), *i*∈{1, …, *n*}, a set of *p* coefficients **β** = (β_1_, β_2_, …, β_*p*_), and matrix of predictor variables

$$X={\left(x_{1},\;x_{2},\ldots ,x_{n}\right)}^{T}=\left(\begin{array}{c} x_{1,1}\;\cdots \;x_{1,p}\\ \vdots \;\ddots \;\vdots \\ x_{n,1}\;\cdots \;x_{n,p} \end{array}\right)$$

For a given λ_1_, λ_2_ ≥ 0, elastic-net regularization uses a criterion based on

$$\begin{array}{l} L\left(\lambda_{1},\lambda_{2},\beta \right)={\left\|y-X\beta \right\|}_{2}^{2}+\lambda_{2}{\left\|\beta \right\|}_{2}^{2}+\lambda_{1}{\left\|\beta \right\|}_{1}\\ \;{\left\|\beta \right\|}_{2}=\sqrt{\sum_{j=1}^{p}\beta_{j}}\\ \;{\left\|\beta \right\|}_{1}=\sum_{j=1}^{p}\left|\beta_{j}\right| \end{array}$$

Thus, the set of coefficient estimates $\hat{\beta }$ are calculated as

$$\begin{array}{l} \hat{\beta }=\underset{\beta }{\text{argmin}}\{L\left(\lambda_{1},\lambda_{2},\beta \right)\}=\underset{\beta }{\text{argmin}}\{{\left\|y-X\beta \right\|}_{2}^{2}+\lambda_{2}{\left\|\beta \right\|}_{2}^{2}\\ \;+\lambda_{1}{\left\|\beta \right\|}_{1}\} \end{array}$$

Given $\alpha =\frac{\lambda_{1}}{\lambda_{1}+\lambda_{2}}$, solving for $\hat{\beta }$ is equivalent to optimizing $\hat{\beta }=||\text{y}-\text{X}\beta |{|}_{2}^{2}$, for $\alpha ||\beta |{|}_{2}^{2}+\left(1-\alpha \right)||\beta |{|}_{1}\leq k,\;for\;some\;k$. In the construction of this elastic net, $\alpha ||\beta |{|}_{2}^{2}+\left(1-\alpha \right)||\beta |{|}_{1}$ is considered as the elastic net penalty, representing a combination of the penalties used in ridge and lasso regression methods. In the situation where α = 1, the elastic net is equivalent to basic ridge regression. For α = 0, the approach becomes lasso regression (Zou and Hastie, 2005).

GeneQC utilizes the elastic-net regularization method (Zou and Hastie, 2005) with default α = 0.5 to develop a regression model for the calculation of D-scores. Here, elastic-net regularization is used to properly perform the variable selection, while simultaneously fitting a sufficient model to the provided data (Figure 1D). This approach also accounts for potential serious multicollinearity issues which were detected in some of the test data and prevents overfitting of the regression model (Zou and Hastie, 2005). The set of calculated D-scores represents the mapping uncertainty for each annotated gene and is provided to give researchers an idea of how reliable their initial read mappings are. A higher D-score represents more mapping uncertainty, and thus a less reliable expression estimate.

#### Mixture model fitting

Based on the calculated sets of D-scores through above investigations during GeneQC development, there are apparent underlying distributions for these scores, intuitively representing levels of mapping uncertainty. For this purpose, extensive mixture model fitting is included within GeneQC to best fit a mixture model distribution with three sub-distributions to each set of D-scores (Figure 1E).

Our mixture model fitting process involves *k*-means initialization with randomized initial grouping. Cluster means, μ_*i*_, are then calculated for each of the *k* clusters, followed by two iterative steps: (1) reassignment of data points to the cluster with the lowest distance between a data point and cluster mean, and (2) recalculation of cluster centers. This process is continued until achieving the minimum within-cluster sum of squares:

$$\begin{array}{l} \underset{\text{k}}{\text{argmin}}\overset{k}{\underset{i=1}{\sum }}\underset{x\in K_{i}}{\sum }||x-\mu_{i}|{|}^{2} \end{array}$$

After initialization using the *k-means* process defined above, the *EM-algorithm* is implemented to find the best fitting distributions. Based on our preliminary investigations into the D-score development, we have selected two underlying distributions for this purpose: Gamma and Gaussian. Specifically, it is assumed that each set of D-scores can be expressed as a mixture model distribution given by

$$\begin{array}{l} P\left(X|\theta \right)=\underset{k}{\sum }\beta_{k}Y_{k}\left(X|\theta_{k}\right) \end{array}$$

with β_*k*_ representing the weighting parameter of the *k*^*th*^ component, *Y*_*k*_ representing the probability density function of the *k*^*th*^ component of the mixture model, and θ_*k*_ representing the parameters of the *k*^*th*^ component. Considering the Gaussian distribution scenario, *Y*_*k*_(*X*|θ_*k*_) is $N\left(X|\mu_{k},\;\sigma_{k}^{2}\right)$. In this case,

() $$\begin{array}{l} \;MLE(\mu_{k})={\hat{\mu }}_{k}=\frac{\sum_{j}^{N_{k}}x_{j,k}}{N_{k}}\\ \;MLE\left(\sigma_{k}^{2}\right)={\hat{\sigma }}_{k}^{2}=\frac{\sum_{j}^{N_{k}}{\left(x_{j,k}-\mu_{k}\right)}^{2}}{N_{k}}\\ \;\beta_{k}=\frac{N_{k}}{N} \end{array}$$

where *x*_*j, k*_ is the *jth* data point in component *k*, *N*_*k*_ is the number of data points in cluster *k* and *N* is the total number of data points (i.e., $\underset{k}{\sum }N_{k}=N$). After this initialization step, the algorithm proceeds to the Expectation (E) step. In this step, for each data point (i.e., each D-score from this dataset) the posterior probability of containment within each cluster *k*_*i*_ is generated by

$$\begin{array}{l} P\left(x_{j}\in k_{i}|x_{j}\right)=\frac{P\left(x_{j}|x_{j}\in k_{i}\right)\;P\left(k_{i}\right)}{P\left(x_{j}\right)}=\frac{N\left(x_{j}|{\hat{\mu }}_{k},{\hat{\sigma }}_{k}^{2}\right)\left(\frac{N_{k}}{N}\right)}{\sum_{k}\beta_{k}N\left(x_{j}|{\hat{\mu }}_{k},{\hat{\sigma }}_{k}^{2}\;\right)}\\ =\frac{\beta_{k}N\left(x_{j}|{\hat{\mu }}_{k},{\hat{\sigma }}_{k}^{2}\;\right)}{\sum_{k}\beta_{k}N\left(x_{j}|{\hat{\mu }}_{k},{\hat{\sigma }}_{k}^{2}\;\right)} \end{array}$$

After this Expectation step, the Maximization step again calculates parameters ${\hat{\mu }}_{k},{\hat{\sigma }}_{k}^{2}\;$for each component *k*. Based on the previous step,

$$\begin{array}{l} {\hat{\mu }}_{k}=\frac{\sum_{j=1}^{N}P\left(x_{j}\in k_{i}|x_{j}\right)x_{j}}{\sum_{j=1}^{N}P\left(x_{j}\in k_{i}|x_{j}\right)}\\ {\hat{\sigma }}_{k}^{2}=\;\frac{\sum_{j=1}^{N}P\left(x_{j}\in k_{i}|x_{j}\right){\left(x_{j}-{\hat{\mu }}_{k}\right)}^{2}}{\sum_{j=1}^{N}P\left(x_{j}\in k_{i}|x_{j}\right)}\\ \beta_{k}=\frac{\sum_{j=1}^{N}P\left(x_{j}\in k_{i}|x_{j}\right)}{N} \end{array}$$

These parameter estimates are then used as the parameters for the next Expectation step, through which this process iteratively continues until convergence, i.e., no significant improvement in the log-likelihood is achieved from the previous iteration. This process is implemented iteratively to quickly generate a series of mixture model distributions for both Gamma and Gaussian distributions.

The optimally fitted mixture model is determined using a Bayesian Information Criterion (BIC) with a penalization based on the number of distributions is used to determine the best-fitting distribution. The BIC for a mixture distribution *K* is based on the number of sub-distributions *k*, the number of data points *n*, and the log likelihood $\hat{L}$.

$$\begin{array}{l} BIC\left(K\right)=-2klog\left(n\right)-2\hat{L} \end{array}$$

#### Mapping uncertainty categorization

The best fitting mixture model is then used to separate each D-score into a category representing the severity of mapping uncertainty, thus indicating the mapping uncertainty categorization for each gene (Figure 1F). The categorizations are based on the intersections of the density functions representing the mixture model fitting. If the Gaussian distributions provide the minimal BIC, the categorization cutoffs are calculated as

$$\begin{array}{l} x=-\left(\frac{\mu_{i+1}\sigma_{i}^{2}-\mu_{i}\sigma_{i+1}^{2}}{\sigma_{i+1}^{2}-\sigma_{i}^{2}}\right)\pm \sqrt{\left(\frac{2\sigma_{i}^{2}\sigma_{i+1}^{2}\cdot \ln\left(\frac{\sigma_{i+1}^{2}}{\sigma_{i}^{2}}\right)-\mu_{i}^{2}\sigma_{i+1}^{2}+\mu_{i+1}^{2}\sigma_{i}^{2}}{\sigma_{i+1}^{2}-\sigma_{i}^{2}}\right)+{\left(\frac{\mu_{i+1}\sigma_{i}^{2}-\mu_{i}\sigma_{i+1}^{2}}{\sigma_{i+1}^{2}-\sigma_{i}^{2}}\right)}^{2}} \end{array}$$

for *i* ∈ {1, 2}.

For Gamma distributions providing the minimal BIC, a closed form solution of the density function intersections does not exist. To accommodate this, an estimation approach is utilized. The cutoffs are calculated as the mean value of the maximum sequence element for which sub-distribution *i* has a higher probability density value than it does for sub-distribution *i* + 1 and the minimum sequence element for which sub-distribution *i* + 1 has a higher probability density value than it does for sub-distribution *i*, i.e.,

$$\begin{array}{l} mean\left(\underset{x}{\text{argmax}}\left\{f_{i}\left(x\right)>f_{i+1}\left(x\right)\right\},\;\underset{x}{\text{argmin}}\left\{f_{i}\left(x\right)<f_{i+1}\left(x\right)\right\}\right)\\ x\in \left\{a_{n}|\;\underset{x}{\text{argmax}}f_{i}\left(x\right)\leq a_{n}\leq a_{n+1}\leq \underset{x}{\text{argmax}}f_{i+1}\left(x\right)\right\} \end{array}$$

resulting in two cutoff values.

Due to the nature of mapping uncertainty and the lack of current approaches to evaluate this concept, we have included an alternative likelihood value, for the first time, as a proposed method of evaluating the mapping uncertainty categorizations computationally. This value based on the posterior probabilities of the other distributions is provided to represent the certainty of the gene ID belonging to that category. This value (*s*_*d*_) is computed as the maximum posterior probability of the D-score belonging to any other categorization distribution.

$$\begin{array}{l} s_{d}=\max\left\{1-F_{i-1}\left(d\right),\;F_{i+1}\left(d\right)\right\} \end{array}$$

where *i* is the distribution for which *d* is categorized, and *F*_*j*_ represents the cumulative distribution function of distribution *j*.

## Results

### GeneQC output

The final output of GeneQC includes the three extracted features (named D_1_, D_2_, and D_3_), D-score, mapping uncertainty categorization, and alternative likelihood for each annotated gene. This information is combined into a concise table to provide users with all relevant information related to the mapping uncertainty of their read alignment data, allowing them to make informed decisions about further and continued analysis. An example of the output file from *Vitis vinifera* can be found in Table 2. For each annotated gene, the D-score indicates the severity of mapping uncertainty for that particular gene in this particular RNA-Seq data. A higher D-score indicates a higher level of mapping uncertainty, with maximum levels of mapping uncertainty occurring around 0.5 for most samples. Genes with relatively high D-scores have mapping uncertainty issues resulting in potentially unreliable expression estimates (i.e., the High category). Whereas, genes with D-scores close to 0 have little to no mapping uncertainty, and therefore have reliable expression estimates (i.e., the Low and Medium categories).

**Table 2:** GeneQC example output.

**Gene ID**	**D1**	**D2**	**D3**	**D-score**	**Category**	**Alternative likelihood**
gene17958	1439.981	0.022727	1.041393	0.022765	Low	0.106445
gene29138	228	1	0.69897	0.509935	High	0.012702
gene17991	2560	1	0.477121	0.498094	High	0.015754
gene24080	321.9987	0.005017	2.060698	0.020863	Low	0.10397
gene23209	365	0.0224	1.78533	0.027916	Low	0.113361
gene420	157	0.04878	0.954243	0.033132	Low	0.120682
gene15973	691.9874	0.7809523	0.47712125	0.39143804	Medium	2.15E-54
gene24933	855	1	0.477121	0.499807	High	0.015276
gene26458	4864	1	0.477121	0.495779	High	0.016419

Source code and implementation instructions can be found on the GeneQC web server at http://bmbl.sdstate.edu/GeneQC/home.html. Additionally, example data for seven analyzed species can be downloaded on this server, including all reference genomes, annotations, original raw data, and outputs from both the feature extraction and modeling portions of GeneQC. An in-depth tutorial for application instructions can also be found on this site.

### Implementation and application of GeneQC results

GeneQC has four main applications in RNA-Seq analyses. (1) Users can take the D-score and categorization results from an entire dataset to evaluate the alignment quality of their data or to determine how severe the overall mapping uncertainty is within their RNA-Seq datasets. This process would involve displaying the set of D-scores in some visualization technique, such as a boxplot, violin plot, or histogram. Displaying the D-scores in this format would allow for users to determine if the overall alignment quality is sufficient to continue analysis or if it requires further evaluation using a re-alignment method. It is expected that there will be high D-scores for some genes; however, a large portion of data having high D-scores would indicate severe problems with alignment requiring further analysis. (2) Users can use D-scores and mapping uncertainty categorizations to evaluate the reliability of their downstream analyses, such as differential gene expression results. If users have identified a particular set of genes that are differentially expressed, it would be of interest to evaluate the reliability of the expression estimates from which those comparisons were made. Genes identified as differentially expressed having high mapping uncertainty levels—either through D-scores or categorization—would be less reliable than the differentially expressed genes that have low mapping uncertainty. (3) GeneQC can be used to directly compare the severity of mapping uncertainty between samples or even between species. This application method is used in section GeneQC Application: Analysis of Seven Plant and Animal Species to demonstrate which species have relatively high levels of mapping uncertainty and to determine which characteristics or features could be affecting this issue. In particular, identification of characteristics impacting mapping uncertainty for a single species could provide information that would assist in re-alignment processes. (4) GeneQC can be used to perform large-scale comparisons of alignment tools using real data. Currently, comparisons of alignment tools require either simulated data which cannot accurately replicate the complexities within real RNA-Seq data, or they rely on small-scale real data, which has implicit biases that may favor one tool. GeneQC allows for the large-scale comparisons of alignment methods with complex data of any species.

### GeneQC application: analysis of seven plant and animal species

**Figure 3:**
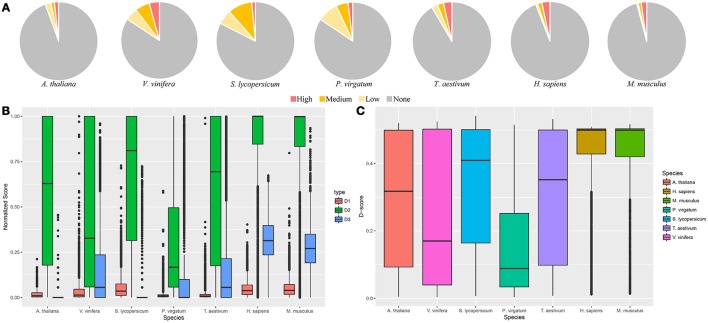
**G**eneQC application. The results related to the analysis of seven datasets representing five plant and two animal species. **(A)** Categorizations for the level of mapping uncertainty per gene are shown relative to all categorizations. **(B)** Boxplots for the three extracted features of each gene are shown for each analyzed sample. D_1_, D_2_, and D_3_ represent the sequence similarity, proportion of shared MMR, and degree weight, respectively. Each value is shown normalized between 0 and 1. Only genes with mapping uncertainty are displayed. **(C)** Derived D-scores for each gene are shown by species, as calculated from the three features in **(B)**. Higher D-scores represent higher levels of mapping uncertainty.

In order to display the use of GeneQC, one dataset from each of the seven species were investigated for multi-mapping issues (Table 3). Based on this analysis, it is evident that plant samples tend to have higher proportions of genes with mapping uncertainty than animal samples (Figure 3A). These results correlate with the fact that plant genomes tend to have higher levels of duplication, which is a strong contributing factor to mapping uncertainty. While *H. sapiens* and *M. musculus* have lower proportions of genes with mapping uncertainty than the plant samples, the proportion of genes with high mapping uncertainty of all the genes with mapping uncertainty is much higher. Plant species exhibited mapping uncertainty in an average of 12.6% of genes across the five species, whereas animal species exhibited this issue in an average of 5% of genes (Supplementary Files S2, S3). However, over half of the genes with mapping uncertainty in the animal samples fall into the “High” categorization, while only around one-fifth of genes with mapping uncertainty from plant samples fall into this category. The contributing factors to the higher proportion of “High” categorized genes for animal samples can be seen when looking at the three extracted features for each species.

**Table 3:** GeneQC analysis of seven species.

**Species**	**Sample ID**	**Mean D_1_**	**Mean D_2_**	**Mean D_3_**	**Mean D-score**
*A. thaliana*	SRR3305038	0.02	0.58	0.01	0.29
*V. vinifera*	SRR2080995	0.04	0.46	0.16	0.24
*S. lycopersicum*	SRR5274891	0.06	0.66	0.04	0.33
*P. virgatum*	SRR5188171	0.01	0.32	0.09	0.16
*T. aestivum*	ATW_AAOSW_6_2 _B06BTABXX.IND12	0.02	0.60	0.15	0.31
*H. sapiens*	SRR6029567	0.05	0.84	0.32	0.43
*M. musculus*	SRR6111161	0.06	0.84	0.28	0.42

*This table shows the sample ID and relevant metrics for each of the seven datasets analyzed. Mean values for D_1_, D_2_, D_3_, and D-score are calculated based on the genes that exhibit some level of mapping uncertainty, and D_1_, D_2_, and D_3_ were normalized for comparison*.

The analysis results for the three features and calculated D-scores for genes with some level of mapping uncertainty are displayed in Figures 3B,C, respectively. Both *H. sapiens* and *M. musculus* display higher levels of sequence similarity (D1), shared MMR proportion (D2), and degree (D3) than what is generally exhibited in the analyzed plant species. These relatively high values for each feature led the higher D-scores, translating to a higher measure of mapping uncertainty in the animal samples compared with the plant samples. Mean D-score for *H. sapiens* and *M. musculus* are 0.43 and 0.42, respectively. These average values are much higher than those for the analyzed plant samples, which are 0.29, 0.24, 0.33, 0.16, and 0.31 for *A. thaliana, V. vinifera, S. lycopersicum, P. virgatum*, and *T. aestivum*, respectively.

## Conclusion

GeneQC is a tool used to investigate the prominent issue of mapping uncertainty in modern RNA-Seq analysis through the combination of feature extraction and machine learning methods. Oversight in the quality of derived gene expression estimates based on mapping results can have drastic consequences for all downstream analyses and read mapping uncertainty is a significant cause of problems in further analysis. While read mapping has been accepted as sufficient, entirely ignoring the possibility of poorly mapped reads used for further analysis can have detrimental effects on all manner of RNA-Seq studies. As demonstrated in our analysis of 95 RNA-Seq datasets, the problem of mapping uncertainty is prominent and is displayed directly in the gene expression estimates. GeneQC can provide insight into the severity of this issue for each annotated gene along with a statistical evaluation framework. It utilizes feature extraction, elastic-net regularization, and mixture model fitting to provide researchers with a sense of the quality of gene expression estimates resulting from the read alignment step. GeneQC provides sufficient information for researchers to make more well-informed decisions based on the results of their RNA-Seq data analysis and to plan further analyses to address mapping uncertainty.

The application of GeneQC on the seven analyzed datasets display some interesting differences between plant and animal samples. Fewer genes displayed mapping uncertainty in the animal samples, while a higher proportion of these genes were categorized as “High.” Alternatively, a much higher proportion of plant genes displayed mapping uncertainty, but more of these genes had moderate to low mapping uncertainty, relative to genes from animal samples. Both of these scenarios display the severity of mapping uncertainty in modern RNA-Seq analyses. High mapping uncertainty displayed in animal samples can lead to very biased expression estimates over fewer genes, while moderate levels of mapping uncertainty on a wider scale as displayed in plant species can cause widespread expression estimate biases on a lesser scale.

## Discussion

In addition to the direct provisions of GeneQC, interpretations of the coefficients allow for a further examination of the specific features contributing the mapping uncertainty. This will allow for further analysis and re-alignment strategies to be developed to the specific characteristics of the dataset. We are currently using this information to develop a computational tool capable of performing re-alignment of reads currently aligned to genes with high D-scores with the purpose of assisting researchers in the correction of mapping uncertainty. In the future, GeneQC will be integrated into a web server that applies this tool and associated re-alignment tools to perform large-scale RNA-Seq analyses on human, plant, and metagenome datasets. This application will allow for ease-of-use and collection of more data to support research with significant MMR issues.

Additionally, further exploration of machine learning approaches, both supervised and unsupervised, will be explored with respect to their applicability in detecting mapping uncertainty. Large-scale use of simulated data for multiple species will provide a direct indication of the actual expression level, which can be compared with the expression estimate from various high-performing and widely-used alignment tools. The various machine learning methods can then be used to detect mapping uncertainty for each tool, with performance comparisons being derived from the correlation between the predicted mapping uncertainty level from the machine learning algorithm and the difference between actual and estimated expression for each gene. A determination for the best-performing method will be based on the highest correlation and may be alignment tool-specific.

## Author contributions

QM conceived the basic idea and designed the analysis. AM, XC, YZ, CW, and QM contributed to development of feature extraction conceptualization, methods, and implementation. AM, SG, JX, and QM contributed to machine learning modeling conceptualization, methods, and implementation. AM, YZ, CW, and QM contributed to the development and maintenance of GeneQC website. AM, SG, and QM contributed to the manuscript development and writing. All authors contributed to the manuscript revisions, read and approved the final version of the manuscript.

### Conflict of interest statement

The authors declare that the research was conducted in the absence of any commercial or financial relationships that could be construed as a potential conflict of interest.
